# Factors associated with Institutional delivery service utilization among mothers in Bahir Dar City administration, Amhara region: a community based cross sectional study

**DOI:** 10.1186/1742-4755-11-22

**Published:** 2014-03-14

**Authors:** Gedefaw Abeje, Muluken Azage, Tesfaye Setegn

**Affiliations:** 1 Public Health Department, College of Medicine and Health Sciences, Bahir Dar University, Bahir Dar, Ethiopia

## Abstract

**Background:**

High maternal mortality is a continued challenge for the achievement of the fifth millennium development goal in Sub-Saharan African countries including Ethiopia. Although institutional delivery service utilization ensures safe birth and a key to reduce maternal mortality, interventions at the community and/or institutions were unsatisfactorily reduced maternal mortality. Institutional delivery service utilization is affected by the interaction of personal, socio-cultural, behavioral and institutional factors. Therefore this study was designed to assess factors associated with institutional delivery service use among mothers in Bahir Dar city administration.

**Methods:**

A community based cross sectional study was conducted in Bahir Dar City administration Northwest of Addis Ababa, Ethiopia. Four hundred eighty four mothers were included in the study. Data were collected by trained female data collectors. Descriptive statistics, binary and multivariable logistic regression analyses were computed. Statistical significance was considered at p < 0.05 and the strength of statistical association was assessed by odds ratios (OR) with 95% confidence intervals.

**Result:**

In this study, 78.8% of women gave birth to their current child at health institution. The multivariable logistic regression showed that, attending primary education (AOR = 4.7[95% CI:1.3-16.7], secondary education (AOR = 3.5[95% CI:1.1-10.7]), age at first marriage; first time marriage at 15–19 years (AOR = 5.4[95% CI:2.0-15.0]) and first time marriage at 20–24 years (AOR = 5.0[95% CI:1.5-16.8] and gestational age at first ANC visit (first trimester) (AOR = 5.3[1.3-22.2]) and second trimester (AOR = 2.8[95% CI:0.7-11.]) were independent factors affecting institutional delivery service utilization.

**Conclusion:**

In this study, institutional delivery service utilization is optimal, urban mothers were more likely to practice institutional delivery. This study indicated that age at first marriage, educational status of the women and gestational age at first ANC visit are independent predictors of delivery service utilization. Hence, intensifying education for women and behavior change communication (BCC) interventions to increase early initiation and up-take of ANC service use in the first trimester and delaying marriage are recommended to promote institutional delivery service utilization.

## Introduction

Maternal mortality is a global problem [1]. The majority of maternal health complications and deaths occurred in low and middle income countries (LMIC) where three quarters of the deaths are due to direct obstetric complication [1-3].

Institutional delivery service utilization is one of the key and proven interventions to reduce maternal death. It ensures safe birth, reduce both actual and potential complications and maternal death and increase the survival of most mothers and newborns. But most deliveries in developing countries occur at home without skilled birth attendants [4-9]. Many low and middle income countries tried their best to optimize key and effective maternal health interventions to improve maternal health [10]. But the progress made in reducing maternal deaths was very far from the Millennium Development Goal (MDGs) targets. It was very slow in African and South Asian countries [6,7,11].

Home delivery is common in many developing countries including Ethiopia. For example, 42.0% of women in Malawi [12], 69% in Nepal [13], 70% in Zaria (Northern Nigeria) [14], 74% in Pakistan [15] and 87.6% in Eastern Burma [16] gave birth at home. Similarly, 81.8% of women in Dodota district, [17], 95.9% in Tigray [18], and 87.7% of women in Arsi [19] reported that they gave birth at home. In Amhara region, 89.3% of mothers gave birth at home which is the fourth highest home delivery rate among the nine Ethiopian regions [5]. Socio-economic, socio-demographic, ANC attendance and health services related factors were factors associated with institutional delivery service utilization in studies conducted in Africa (including Ethiopia) and Southern Asia. Not only maternal variables, husbands’ personal, socio-demographic and wealth related variable were associated with delivery service use [12-14,20,21].

Ethiopia is a Sub-Saharan African country with the highest level of maternal mortality ratio mortality ratio (676 mothers die per 100,000 live birth) [5,22,23]. Although not significant, the three consecutive Ethiopian Demographic Health Surveys (EDHSs) showed decreasing trend of maternal mortality ratio. This insignificant decline of maternal mortality ratio might be due to the non use of institutional delivery services associated with residence, educational status, knowledge and ANC attendance [24].

Therefore, in recognition of the national burden of maternal mortality and the urgency to achieve the Millennium Development Goal 5 (MDG5), the government of Ethiopia is committed to improve maternal health with a target of reducing maternal mortality ratio (MMR) to 267/100,000 live births through multi-pronged approaches including provision of free delivery service [25]. But institutional delivery service use was low and majority of women in Ethiopia have been giving birth at home. This might be due to lack of research evidences on important determinants of institutional delivery service use. Therefore, this study was intended to assess factors associated with institutional delivery service use among mothers in Bahir Dar City Administration, Amhara region.

## Methods

### Study setting and sample

Community based cross sectional study was conducted in Bahir Dar City administration from June 15-July 15, 2012 on women who gave birth 12 months before the study period. The city administration has 18 (9 urban and 9 rural) kebeles. Bahir Dar town is the capital city of Amhara National Regional state and located 565 KMs Northwest of Addis Ababa, Ethiopia. The total population of the town (without including the rural Kebeles) is estimated to be 180,174 of which, 93,014 are females [24,26]. There were three hospitals (one governmental referral hospital and two private hospital), 8 health centers (4 in urban, 4 in rural), 2 nongovernmental clinics, 34 private clinics and 10 health posts that provide promotive, preventive, curative and rehabilitative services to the community. There were a total of 693 health professionals working at different health institutions [27].

Sample size was determined using single population proportion formula taking the prevalence of institutional delivery service use in Amhara region (10.2%) [5] with 95% confidence level and 4% margin of error. Since multistage sampling technique was used, the calculated sample size (n = 220 mothers) was multiplied by a design effect of 2 and 10% for non response was added, the final sample size was 484 mothers.

The total kebeles (smallest administrative unit) were divided as rural and urban kebeles. Four kebeles (2 rural and 2 urban) were selected randomly. The total sample size was allocated proportionally to the number of selected kebeles. Then, mothers who gave birth 12 months prior to the date of the survey were systematically selected and included. When there were more than one eligible mother in the selected household, lottery method was used to select either of the mother.

### Measurements

Data were collected using structured, interviewer administered Amharic (local language) version questionnaire. Socio-demographic, obstetric and maternal data were collected. Home delivery was considered when a mother reported birth at home (other than health institution) to her recent delivery. Data were collected through face to face interview. Data collectors and supervisors were trained for three days on the objectives of the study. To ensure data quality, proper designing, translation (to the local language) and pre-testing of the research tool were done. The filled data was checked for completeness on daily basis and feedback was given to data collectors on the next morning.

### Data analysis

Data were checked for completeness and inconsistencies, entered, coded, cleaned and analyzed using SPSS version 16.0. Descriptive statistics was computed to determine proportions. Binary and multivariable logistic regression analyses were carried out to identify factors associated with institutional delivery service use. All tests were two-sided and statistical significance was considered at p < 0.05. The strength of statistical association was assessed by odds ratios (OR) with 95% confidence intervals.

### Ethical clearance

Letter of ethical approval was received from Institutional Review Board (IRB) of Bahir Dar University. The purpose of the study, potential risk and benefits and rights of participants were explained. Verbal consent was obtained from the participants. The participants were assured about the confidentiality of the information they provided.

## Result

### Socio-demographic characteristics

In this study, a total of 484 mothers were reached and 481 mothers were included in the analysis which made the response rate 99.4%. Majority (83.4%) of respondents were urban mothers. The mean (±SD) age of mothers was 27.9 (±6.0) years. Ninety percent (90%) of mothers were married while 2.5% were singles (never married). Majority (65.1%) of mother were house wives. Forty six percent (45.9%) of mothers reported that their husbands were private employee or have engaged in running their own business. Thirty four percent (33.6%) of mothers have attended secondary and above grades while 44.1% of mother reported that their husbands have attended secondary and above grades (Table 1).

**Table 1:** Socio-demographic characteristics of women and husbands, Bahir Dar City Administration; Northwest Ethiopia, 2013

**Variables**	**Frequency**	**Percent**
**Residence**		
Urban	401	83.4
Rural	80	16.6
**Age of mother (current)**		
15-19	15	3.1
20-24	137	28.5
25-29	151	31.4
30-34	81	16.8
35+	97	20.2
Mean (±SD) age (years)	27.9 (±6.0)	
**Marital status**		
Married	437	90.9
Never married	12	2.5
Divorced/widowed/separated	32	6.6
**Religion**		
Orthodox	416	86.5
Catholic and protestant	65	13.5
**Ethnic group**		
Amhara	459	96.4
Tigre	10	2.1
Others^★^	7	1.5
**Occupation of mothers**		
House wife	311	65.1
Government employee	56	11.7
Private employee/business	81	16.9
Other^$^	30	6.3
**Occupation of husbands**		
Government employee	120	26.6
Private employee/business	207	45.9
Other*	124	27.5
**Educational level of mothers**		
Illiterate	180	37.6
Read and write	66	13.8
Primary education	72	15.0
Secondary and above	161	33.6
**Educational level of husbands**		
Illiterate	100	21.9
Read and write	71	15.6
Primary education	84	18.4
Secondary and above	201	44.1

^★^Awi, Oromo, ^$^daily labour, students, jobless, *daily labour, jobless, students, taxi drivers.

### Obstetric and maternal characteristics

In this study, the mean (±SD) age at first marriage and age at first pregnancy were 19.3(±4.7) and 21.8(±4.5) years respectively. Forty nine percent (49%) (cumulative proportion) of participants got married at the age of ≤19 years. Thirty one percent (31.4%) of the mothers had got their first pregnancy at their early age (≤19 years). Of the total birth, 19.0% were from unplanned pregnancies. Only 55.9% of mothers had reported antenatal care visits at their first trimester for current birth (Table 2).

**Table 2:** Obstetric and maternal characteristics of women, Bahir Dar City Administration; Northwest Ethiopia, 2013

**Age at first marriage**	**Frequency**	**Percent**
<15	97	20.2
15-19	142	29.5
20-24	168	34.9
25+	74	15.4
Mean (±SD) age at first marriage	19.3 (±4.7) years	
**Age at first pregnancy**		
<15	32	6.7
15-19	119	24.7
20-24	208	43.2
25+	122	25.4
Mean (±SD) age at first pregnancy	21.8 (±4.5) years	
**Age at the last pregnancy**		
<15	7	1.5
15-19	27	5.6
20-24	160	33.3
25+	287	59.7
Mean(±SD) age at current pregnancy	26.9(±6.3) years	
**Number of pregnancy(gravidity)**		
<3	329	68.4
≥3	152	31.6
**Last pregnancy**		
Planned	389	81.0
Unplanned	91	19.0
**Gestational age at first ANC visit**		
First trimester	229	55.9
Second trimester	167	40.7
Third trimester	14	3.4
**Number of live births (parity)**		
<3	340	70.7
≥3	141	29.3

### Factors associated with institutional delivery service utilization

In this study, although 83.4% mothers were urban respondents, the prevalence of institutional and home delivery was 78.8% and 21.2% respectively. Among the socio-demographic variables, age, marital status, occupation and educational status of mothers were statistically associated with institutional delivery service use. Similarly, occupation and educational status of husbands showed statistically significant association with institutional delivery service utilization. After adjusting for potential confounder, attending primary education (AOR = 4.7[95% CI: 1.3-16.7] and secondary education (AOR = 3.5[95% CI: 1.1-10.7]) were independent social predictors of institutional delivery service utilization (Table 3).

**Table 3:** Socio-demographic factors associated with institutional delivery service utilization among reproductive age women, Bahir Dar City Administration; Northwest Ethiopia, 2013

**Variables**	**Place of delivery**		**COR [95% CI]**	**AOR [95% CI]**
	**HI**^**♦**^**(%)**	**Home (%)**		
**Residence**				
Urban	364 (90.8)	37 (9.2)	42.6 [22.1-82.1]*	-
Rural	15 (18.8)	65 (81.2)	1.0	-
**Current age of mothers**				
15-19	7 (46.7)	8 (53.3)	1.0	1.0
20-24	109 (79.6)	28 (20.4)	4.5 [1.5-13.3]*	4.9 [0.72-33.9]
25-29	119 (78.8)	32 (21.2)	4.3 [1.4-12.6]*	5.6 [0.82-38.9]
30-34	64 (79.0)	17 (21.0)	4.3 [1.4-13.5]*	3.8 [0.53-27.2]
35+	80 (82.5)	17 (17.5)	5.4 [1.7-16.8]*	3.9 [0.53-28.7]
**Marital status**				
Never married	6 (50.0)	6 (50.0)	1.0	
Married	347 (79.4)	90 (20.6)	3.9 [1.2-12.2]*	
Divorced/widowed/separated	26 (81.2)	6 (18.8)	4.3 [1.0-18.3]	-
**Occupation of mothers**				
House wife	229 (73.6)	82 (26.4)	1.0	1.0
Government employee	54 (96.4)	2 (3.6)	9.7 [2.3-40.5]*	1.7 [0.2-16.0]
Private employee/business	71 (87.7)	10 (12.3)	2.5 [1.3-5.20]*	1.4 [0.5-3.8]
Other^$^	22 (73.3)	8 (26.7)	1.0 [0.4-2.3]	2.5 [0.8-8.4]
**Occupation of husbands**				
Private employee/business	183 (88.4)	24 (11.6)	1.0	1.0
Government employee	116 (96.7)	4 (3.3)	3.8 [1.3-11.2]*	1.4 [0.4-4.9]
Other♢	60 (48.4)	64 (51.6)	0.1 [0.1-.2]	0.1 [0.1-0.3]
**Educational level of mothers**				
Illiterate	101 (56.1)	79 (43.9)	1.0	1.0
Read and write	57 (86.4)	9 (13.6)	5.0 [2.3-10.6]*	1.5 [0.6-4.0]
Primary education	66 (91.7)	6 (8.3)	8.6 [3.5-20.8]*	4.7 [1.3-16.7]*
Secondary and above	154 (95.7)	7 (4.3)	17.2 [7.6-38.8]*	3.5 [1.1-10.7]*
**Educational level of husbands**				
Illiterate	47 (47.0)	53 (53.0)	1.0	1.0
Read and write	52 (73.2)	19 (26.8)	3.1 [1.6-5.9]*	0.9 [0.4-2.2]
Primary education	69 (82.1)	15 (17.9)	5.2 [2.6-10.3]*	1.8 [0.8-4.4]
Secondary and above	193 (96.0)	8 (4.0)	27.2 [12.1-61.1]*	3.1 [0.9-9.8]

^♦^Health Institution, ^$^daily labour, students, jobless, ♢daily labour, jobless, students, taxi drivers, *statistically significant at P < 0.05 (two-tailed), COR = Crude Odds Ratio, AOR = Adjusted Odds Ratio.

In this study obstetric and maternal variables were treated separately to see their association with place of delivery. The binary logistic analysis showed that age at first marriage, number of pregnancies, type of pregnancy (planned Vs unplanned), gestational age at first ANC visit and number of live births showed statistically significant association with institutional delivery service use. But in the multivariable logistic regression analysis, age at first marriage (15–19 years) (AOR = 5.4[95% CI: 2.0-15.0]) and married at 20–24 years (AOR = 5.0[95% CI: 1.5-16.8] and gestational age at first ANC visit (AOR = 5.3[95% CI: 1.3-22.2]) were independently associated factors with institutional delivery service utilization (Table 4).

**Table 4:** Maternal and obstetric factors associated with institutional delivery service utilization among reproductive age women, Bahir Dar City Administration; Northwest Ethiopia, 2013

**Variables**	**Place of delivery**		**COR [95% CI]**	**AOR [95% CI]**
**Age at first marriage**	***HI (%)***	***Home (%)***		
<15	46 (47.4)	51 (52.6)	1.0	1.0
15-19	120 (84.5)	22 (15.5)	6.0 [3.3-11.1]*	5.4 [2.0 -15.0]*
20-24	150 (89.3)	18 (10.7)	9.2 [4.9-17.4]*	5.0 [1.5 -16.8]*
25+	63 (85.1)	11 (14.9)	6.4 [2.9-13.5]*	1.7 [0.4 -7.7]
**Age at first pregnancy**				
<15	14 (43.8)	18 (56.2)	1.0	1.0
15-19	74 (62.2)	45 (37.8)	2.1 [0.9-4.7]	1.2 [0.4-3.8]
20-24	180 (86.5)	28 (13.5)	8.3 [3.7-18.5]*	2.5 [0.6 -10.2]
25+	111 (91.0)	11 (9.0)	12.9 [5.1-33.0]*	2.3 [0.4 -13.6]
**Age at last pregnancy**				
<15	4 (57.1)	3 (42.9)	1.0	
15-19	17 (63.0)	10 (37.0)	1.3 [0.2-6.9]	0.5 [0.0-6.8]
20-24	125 (78.1)	35 (21.9)	2.7 [0.6-12.5]	1.0 [0.1 -10.4]
25+	233 (81.2)	54 (18.8)	3.2 [0.7-14.9]	2.9 [0.3 -31.4]
**Number of pregnancy (gravidity)**				
<3	281 (85.4)	48 (14.6)	3.2 [2.0-5.1]*	0.3 [0.0 -5.6]
≥3	98 (64.5)	54 (35.5)	1.0	1.0
**Last pregnancy**				
Planned	313 (80.5)	76 (19.5)	1.6 [1.0-2.8]*	--
Unplanned	65 (71.4)	26 (28.6)	1.0	--
**Gestational age at first ANC visit**				
First trimester	209 (91.3)	20 (8.7)	10.4 [3.3-32.8]*	5.3 [1.3 -22.2]*
Second trimester	139 (83.2)	28 (16.8)	5.0 [1.6-15.3]*	2.8 [0.7 -11.3]
Third trimester	7 (50.0)	7 (50.0)	1.0	1.0
**Number of live births (parity)**				
<3	290 (85.3)	50 (14.7)	3.4 [2.2-5.3]*	--
≥3	89 (63.1)	52 (36.9)	1.0	--

*significantly associated at p < 0.05 (two tailed) HI = Health Institution, COR = Crude Odds Ratio, AOR = Adjusted Odds Ratio.

## Discussion

This study was designed to assess institutional delivery service use and associated factors among mothers who gave birth in the last 12 months. Although majority of respondent were urban mothers, 21.2% of them gave birth at home of which 81.2% were rural mothers which indicated that home delivery was more prevalent in rural kebeles of the city administration. Ninety eight percent (98.3%) of mothers perceived that institutional delivery service use has benefits. However, only 78.8% of them gave birth in health institution. This finding is consistent with a study done in Addis Ababa city in which 82.3% of women gave birth in health institutions. But this proportion is higher compared with institutional delivery service use among urban mothers at national and regional level (Amhara region) [5]. The reason for this difference might be that this study was conducted after the Ethiopian government started free delivery services at all levels of health facilities.

The prevalence of home delivery in this study was less compared with findings from other developing countries like Malawi [12], Nepal [13], Zaria, Northern Nigeria [14], Pakistan [15], and Eastern Burma [16]. The proportion of home delivery was also low even compared with the national and regional proportion of home delivery [5] and other studies done in Ethiopia [17,18,28]. The reason for this comparatively low proportion of home delivery in our study might be due to the fact that high proportions of urban mothers (83.4%) were included in our study. Additionally, it might be due to high perceived benefit of mothers towards institutional delivery service use (98.3%) and perceived maternal health risks associated with home delivery (95.8%).

The multivariable logistic regression analysis showed that mothers’ educational status (primary and secondary level) was important factor associated with institutional delivery service use in this study. Mothers who attended primary education were 4.7 times more likely to give birth in health institutions compared with illiterate mothers. Similarly, mothers who attended secondary education were 3.5 times more likely to utilize institutional delivery service compared with illiterate mothers (Table 3). In this study, age at first marriage and early ANC visits were also determinant factors of institutional delivery service use. Our study revealed that mothers who married at age 15–19 and 20–24 years were 5.4 and 5 times more likely to give birth in health institutions respectively compared to mothers married at age <15 years. Previous studies conducted in Ethiopia revealed that receiving early and on time ANC advice will prepare mothers for child birth and encourage them to give birth in health institutional [17-19,29].

In this study, only 55.9% of study participants visited ANC at first trimester of their pregnancy. In this study, mothers who had their first ANC visit at their first trimester were 5.3 times more likely to give birth in health institutions while mothers who had their first ANC visit at their second trimester were 2.8 times more likely to give birth in health institutions compared to mothers who made their first ANC visit at their third trimester (Table 4).

The strengths of this study is that interviewers were extensively trained on the ways of facilitating sincere responses from the respondents’ side. On the other hand, mothers might not have reported events correctly due to memory lapse and this could introduce recall biases. We were unable to measure reason for the preference of birth place qualitatively. In this study some confidence intervals were somehow wide due to low number of cases per variables which would affect the precision of estimates. Since the study is cross sectional in its very nature, we could not establish causal relationship of the explanatory and outcome variables. Finally, this study is also limited to those missing data, thus interpretation of the finding shall take the missing data in to account.

## Conclusion

Although institutional delivery service use among women in Bahir Dar city administration was higher compared with the national and regional estimates, higher proportion of women from the rural kebeles of the city administration gave birth at home. This study identified that educational status of mothers, age at first marriage and gestational age at first ANC visit as important predictors of institutional delivery service use among mothers. Mothers who have attended formal education showed better use of institutional delivery service. Early age at first marriage was also significant predictor of institutional delivery service use. Therefore, intensifying women’s education, promoting up take of ANC visit in the first trimester, delaying marriage (preventing early marriage) are recommended interventions to increase institutional delivery service use.

## Competing interest

The authors declare that they have no competing interests.

## Authors’ contributions

GA conceived and designed the study, assisted in analysis and interpretation of the result and critically reviewed the manuscript. MA assisted with the design conception, analysis, and interpretation of the result and reviewed the manuscript critically. TS assisted the study design, analyzed the data, interpreted the result and prepared the manuscript. All authors read and approved the final manuscript.
